# Open-Source Colorimeter

**DOI:** 10.3390/s130405338

**Published:** 2013-04-19

**Authors:** Gerald C. Anzalone, Alexandra G. Glover, Joshua M. Pearce

**Affiliations:** 1 Department of Civil and Environmental Engineering, Michigan Technological University, 1400 Townsend Drive, Houghton, MI 49931, USA; E-Mail: gcanzalo@mtu.edu; 2 Department of Materials Science and Engineering, Michigan Technological University Houghton, 1400 Townsend Drive, Houghton, MI 49931, USA; E-Mail: agglover@mtu.edu; 3 Department of Electrical and Computer Engineering, Michigan Technological University Houghton, 601 M&M Building, 1400 Townsend Drive, Houghton, MI 49931, USA

**Keywords:** open source, open-source hardware, colorimetery, COD, Arduino, RepRap, 3-D printer, open-source sensor, chemical oxygen demand, open-source colorimeter

## Abstract

The high cost of what have historically been sophisticated research-related sensors and tools has limited their adoption to a relatively small group of well-funded researchers. This paper provides a methodology for applying an open-source approach to design and development of a colorimeter. A 3-D printable, open-source colorimeter utilizing only open-source hardware and software solutions and readily available discrete components is discussed and its performance compared to a commercial portable colorimeter. Performance is evaluated with commercial vials prepared for the closed reflux chemical oxygen demand (COD) method. This approach reduced the cost of reliable closed reflux COD by two orders of magnitude making it an economic alternative for the vast majority of potential users. The open-source colorimeter demonstrated good reproducibility and serves as a platform for further development and derivation of the design for other, similar purposes such as nephelometry. This approach promises unprecedented access to sophisticated instrumentation based on low-cost sensors by those most in need of it, under-developed and developing world laboratories.

## 1. Introduction

Free and open-source software (FOSS) is computer software that is available in source code (open source) form and that can be used, studied, copied, modified, and redistributed without restriction, or with restrictions that only ensure that further recipients have the same rights under which it was obtained [1]. FOSS is now the relative standard, with 94% of the World's top 500 super computers, 75% of the top 10,000 websites and 98% of enterprises using open-source software [2–4]. Due to the tremendous success of FOSS development, the concept of open source has spread to areas of inquiry such as education [5,6], appropriate technology for sustainable development (OSAT) [7,8], science [9], nanotechnology [10–12], medicine [13,14], and even hardware [15]. Of the open-source hardware projects, the recent development of low-cost open-source 3-D printers, like the RepRap, has been the most useful for a wide array of research as it has made rapid prototyping accessible to most university laboratories [16–18]. RepRap's open-source and self-replicating nature (approximately 50% of its own parts can be self-printed and it can be assembled largely with off-the-shelf hardware store items and a few specialized electronics that are readily available online [17,18]) makes it an extremely useful platform for open-source fabrication of scientific tools [19]. Since the RepRap's introduction in 2006, the open-source hardware community has rapidly grown and now supports both online businesses solely dedicated to supplying 3-D printers, printer parts and 3-D printing supplies and galleries of open-source designs of both improvements on the printer design but also many other objects including scientific tools, which are hosted on purpose-built sites. These open-source hardware designs sites permit downloading and printing of designs as well as modification and derivation of new designs mimicking long-established open-source software protocols [20]. Recent work has demonstrated that the costs of scientific equipment can be radically reduced by applying open-source principles to their design using a combination of the open-source Arduino electronics prototyping platform and the RepRap 3-D printer [19]. This open-source hardware approach is used here to create an open-source colorimeter.

A colorimeter is a relatively simple sensor device consisting of a light source, sample (cuvette) holder, light intensity sensor and means of controlling the light source and integrating transmitted light intensity. Incident light is generally filtered allowing only a narrow band near the absorbance peak for a given dissolved species. The method requires a blank solution for calibration (zero) and reports results in absorbance units, transmittance or applies the Beer-Lambert law to report results as concentration [21]. Colorimetric methods are used widely in research and industry, including investigating food during storage such as bread [22], chocolate [23] and milk [24]. Colorimeters are used to monitor the levels of nitrates, phosphates, metals and other compounds present in effluent from the manufacture of paper and other commercial goods [25,26]. They have also traditionally been used to estimate the population density of protozoa in a culture and more recently by the medical community to measure the UV radiation exposure of children though skin color changes and to study the aging of bruises [27–29]. One of the more popular applications of a colorimeter is measuring the chemical oxygen demand (COD) for estimating the organic content of waste waters. COD is also a measure included in some water quality indices (WQI) [30]. There are sophisticated and expensive methods to determine COD with high accuracy [31,32], but often at high cost and increased production of waste from the analyses.

This paper provides a methodology for the design, development, and technical validation of an open-source colorimeter using the closed reflux COD method (EPA method 5220D). This approach is evaluated for its potential to reduce the cost of equipment to perform colorimetric COD and the results are discussed to provide conclusions about future development of open-source analytical tools. Whereas a previous open-source colorimeter, the IORodeo Educational Colorimeter Kit [33], was developed for use in the education environment, the instrument presented here is purpose-built to perform this single analysis so as to determine the efficacy of applying the open-source methodology to development of research-grade, bespoke instruments in a research environment. The colorimeter was selected because of its simplicity and utility, making it an ideal instrument to launch an investigation into this methodology.

## 2. Experimental Section

**Figure 1.**
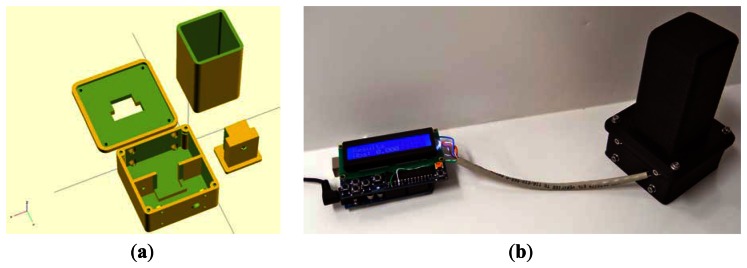
The open-source colorimeter: (**a**) schematic of case design in OpenSCAD, and (**b)** the assembled case with electronics.

The open-source colorimeter case design was wholly completed in OpenSCAD [34], a freely available, open-source, script-based solid modeling software in wide use by the 3-D printing community. The design of the case body is shown schematically in Figure 1(a). The assembled case with electronics is shown in Figure 1(b). The case was printed with black polylactic acid (PLA) media so as to minimize stray light inside the detection area. A RepRap 3-D printer was used to produce the print after slicing the OpenSCAD-produced stereo lithography model (STL) with the open-source Slic3r software [35].

**Figure 2.**
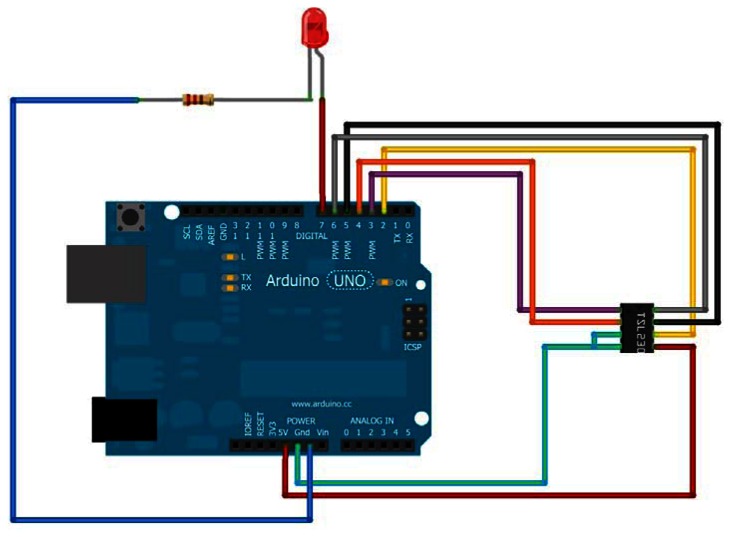
The open-source colorimeter circuit schematic.

The electronics are based upon the open-source Arduino prototyping platform [36], which exposes the digital and analog I/O as well as processing capabilities of an Atmel Atmega microprocessor in a single convenient package. The platform encapsulates the hardware required to interface the Arduino with a host computer and includes a custom bootloader that executes compiled C++ code that can be developed in the Arduino integrated development environment (IDE). The platform is designed to use “shields” or customized electronic boards that can be pressed into place and that typically come with software libraries so as to facilitate integration of board features into the custom code developed by the end user. A display shield incorporating a 16 × 2 character alphanumeric LCD and D-pad button interface was used to navigate and select device functions and display the results of analysis. The shield is also open source, supplied by Adafruit Industries [37], a company dedicated to developing and providing low-cost, innovative and useful open-source electronic solutions. A single 5 mm through-hole LED having an emission peak near 606 nm was used as a light source and a Taos TSL230R light intensity-to-frequency sensor was employed to measure incident light intensity. A total of three discrete electronic components are required for the circuit, as shown schematically in Figure 2. The device's firmware was developed with the Arduino IDE and provides an easy to navigate hierarchical menu system for selection of device functions.

Colorimetric methods are used to determine the concentration of dissolved species, relying on the ability of many ionic species to absorb light of one or more specific wavelengths following the Beer-Lambert law shown in Equation (1):

(1) $$A=-{\text{log}}_{10}(\frac{I}{I_{0}})$$

where A is absorbance (absorbance units), I is the intensity of light passing through unknown, and I_0_ is the intensity of light passing through the blank. The absorbance is related to concentration as shown in Equation (2):

(2) $$A=a_{\lambda }\times b\times c$$

where *a*_λ_ is the molar absorptivity of the species of interest at a certain wavelength, b is the path length of light through solution, and c is the concentration of analyte. The wavelength of light absorbed is an intrinsic property of the analyte and instruments typically measure absorbance of a specific wavelength that is a property of the species of interest.

The closed reflux COD method [38] was selected for evaluating the open-source colorimeter because COD vials were readily available from other research and also due to the availability of LED light sources having peak emission near the 606 nm chromic absorbance peak. The closed reflux colorimetric method used for COD employs potassium dichromate (K_2_Cr_2_O_7_) as oxidant, requires only a small sample size and produces minimal waste. A 2 mL aliquot is added to dichromate solution and allowed to digest at 150 °C for 2 hours. Concentration (mg COD/L) is determined by measuring the increase in absorbance at 606 nm, the absorbance peak for the chromic ion, or determining excess dichromate by measuring the absorbance at 440 nm [38,39].

High range COD digestion vials from the Hach Company (Loveland, CO, USA) were used to digest samples produced during unrelated research activities conducted to develop precision and bias statements for a newly developed analytical procedure. 147 vials were digested and used to conduct this comparison. Digestion was performed with a Hach DRB200 and absorbance was determined by both the OS colorimeter and a commercial Hach DR890 portable colorimeter. Absorbance units were recorded from both units sequentially with five replicate readings from each.

## 3. Results and Discussion

**Figure 3.**
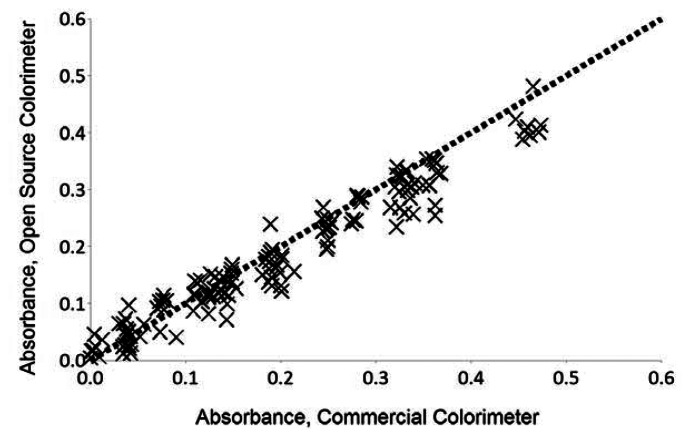
Comparison of the results from the commercial and open-source colorimeter.

A comparison of the results from the commercial and open-source colorimeter are presented graphically in Figure 3. The open-source colorimeter yielded a greater average single sample standard deviation (0.0010 absorbance units) as compared to the commercial colorimeter (0.0002 absorbance units). It should be noted that the standard deviation is not shown in Figure 3 because it is smaller than the data points. However, the open-source colorimeter results were well within (2 mg COD/L) the stated precision of 17 mg COD/L of the Hach method [40]. This indicates that it is suitable for use in any of the standard COD applications within the range investigated. Absorbance determined by the open-source colorimeter is skewed somewhat low compared to the commercial equipment, which is the result of the light source emission spectra having a peak farther away from 606 nm (the LED light source has a dominant wavelength of 620 nm) as compared to the light source or detector range used by the commercial colorimeter. Since the colorimeter was designed to use commercially available COD digestion vials having a stated range of 20 to 1,500 mg/L COD and only side-by-side evaluation of performance was desired, no attempts were made to establish detection limits or linearity.

These results are remarkable considering the open-source colorimeter can be built for one tenth the price (approximately $50) of the least expensive, commercial COD instrument available and two orders of magnitude less than the Hach DR890 that it was compared to in this evaluation. Both the hardware design [41] and software [42] are now freely available online and under the creative commons CC-BY-SA license such that they can be modified and new designs derived.

As the results show, the open-source hardware design approach used here to develop an extremely low-cost COD instrument proved successful. The once onerous learning curve associated with “open source” has largely been overcome due to innovation and rapid development of tools such as the Arduino prototyping platform, the RepRap, and associated software. The entire process of designing, printing and assembling required less than 100 man hours, drew extensively on previously completed open-source work [33,37,43], requiring only moderate literature review and moderate skill levels to design and build. As this project was shared back with the open-source scientific community the time for another group to build their own open-source COD instrument is estimated to be under 10 hours at a cost of approximately US$50.00. Similarly, the time for others to create an open-source colorimeter is also reduced for other applications [22–32] such as: Measuring the concentration of some chemicals in a solution, quantifying observations of biological specimens, growth cultures, food science, quality control in manufacturing, diagnose some diseases, test the concentration of hemoglobin in blood, determining the efficacy of sun protection products, nephelometry for water quality, visibility, and global warming studies to measure global radiation balance, and many other applications. The open-source IORodeo colorimeter kit has already realized some of these applications for the educational environment [44], however, future work is necessary to design focused research tools for all of these applications. A major benefit of the approach shown here is that as both the design files are freely available to replicate this COD instrument in another lab with open-source 3-D printing will take only a few hours. Using this approach on other types of tools such as optics equipment has shown over 97% cost reductions for laboratories [45]. In addition, the OpenSCAD code has been made available, making it easy to redesign the case to test for example alternative sizes or geometries of vials. In the same way, the Arduino software made available here is easily altered for example to adjust integration time, light intensity or sensor sensitivity for another application.

Future work is necessary for this design to realize the full potential of the tool for applications discussed above. In addition, work is needed to make the open-source colorimeter portable, such as the incorporating batteries and solar photovoltaic power. A multi-compartment design that can run more than one type of experiment using the same Arduino and control logic and simply adding additional LEDs and sensors for different types of tests is already being considered. Open-source wireless communication devices are extant, making possible wireless communication between the instrument and a smart phone or tablet, augmenting data recording and analysis capabilities. Modification of the OpenSCAD design may also permit the device to be used in-line for process control or quality assurance/quality control.

As additional research groups begin to freely share the designs of their own open-source sensors, not only can the greater scientific community enjoy the same discounts on equipment, but following the FOSS approach, the equipment will evolve, becoming technically more advanced, easier to use and more useful. It is also likely that the price pressure from the open-source community [19] will drive down costs of commercial versions of the equipment, resulting in a decrease in overall research costs. For example, rapid advancement in 3-D printing technology has already produced plug-and-play 3-D printers at a price point equivalent to or lower than the cost of a television.

To put this work in further perspective, for about the cost of a standard commercial COD instrument a research lab can purchase an open-source 3-D printer and all the parts to make the open-source colorimeter described here. Thus, perhaps most importantly, the ease and low cost of this approach for developing sensor-based tools enables increasingly sophisticated tools to be used in low-funded developing world laboratories, helping to disseminate OSAT for sustainable development. In addition, high-quality open-source scientific hardware, such as the colorimeter described here provides public, non-profit and non-governmental institutions, schools and amateur scientists the tools necessary to conduct real science, while driving down the costs of research tools at our most prestigious corporate, government, and academic laboratories.

## 4. Conclusions and Outlook

The expense of sophisticated research-related sensors and tools has often limited their adoption to a select, well-funded few. This paper provided a methodology for applying the FOSS approach to design and development of an open-source colorimeter; a methodology that eliminates cost as a barrier to adoption and makes the tool available to the broadest possible audience. The performance of the tool produced by this methodology was successfully demonstrated against a much more costly commercial product. An open-source 3-D printable solution for colorimetric determination of COD was successfully demonstrated and the design is now freely available for reuse, derivation and modification.

The outlook for development of scientific-grade instrumentation utilizing the FOSS approach is extremely promising. Inexpensive open-source 3-D printers and free software have put one-off production of highly specialized tools within the grasp of the end user, bypassing historically expensive design and manufacturing steps. Perhaps more importantly, these technologies and methodologies promise heretofore unheard of access to sophisticated instrumentation by those most in need of it, under-developed and developing world laboratories.
